# In Situ Filling of the Oxygen Vacancies with Dual Heteroatoms in Co_3_O_4_ for Efficient Overall Water Splitting

**DOI:** 10.3390/molecules28104134

**Published:** 2023-05-16

**Authors:** Wei Duan, Shixing Han, Zhonghai Fang, Zhaohui Xiao, Shiwei Lin

**Affiliations:** State Key Laboratory of Marine Resource Utilization in South China Sea, School of Materials Science and Engineering, Hainan University, No. 58 Renmin Road, Haikou 570228, Chinalinsw@hainanu.edu.cn (S.L.)

**Keywords:** Co_3_O_4_, oxygen vacancies, in situ filling heteroatoms, electrocatalysts, overall water splitting

## Abstract

Electrocatalytic water splitting is a crucial area in sustainable energy development, and the development of highly efficient bifunctional catalysts that exhibit activity toward both hydrogen evolution reaction (HER) and oxygen evolution reaction (OER) is of paramount importance. Co_3_O_4_ is a promising candidate catalyst, owing to the variable valence of Co, which can be exploited to enhance the bifunctional catalytic activity of HER and OER through rational adjustments of the electronic structure of Co atoms. In this study, we employed a plasma-etching strategy in combination with an in situ filling of heteroatoms to etch the surface of Co_3_O_4_, creating abundant oxygen vacancies, while simultaneously filling them with nitrogen and sulfur heteroatoms. The resulting N/S-V_O_-Co_3_O_4_ exhibited favorable bifunctional activity for alkaline electrocatalytic water splitting, with significantly enhanced HER and OER catalytic activity compared to pristine Co_3_O_4_. In an alkaline overall water-splitting simulated electrolytic cell, N/S-V_O_-Co_3_O_4_ || N/S-V_O_-Co_3_O_4_ showed excellent overall water splitting catalytic activity, comparable to noble metal benchmark catalysts Pt/C || IrO_2_, and demonstrated superior long-term catalytic stability. Additionally, the combination of in situ Raman spectroscopy with other ex situ characterizations provided further insight into the reasons behind the enhanced catalyst performance achieved through the in situ incorporation of N and S heteroatoms. This study presents a facile strategy for fabricating highly efficient cobalt-based spinel electrocatalysts incorporated with double heteroatoms for alkaline electrocatalytic monolithic water splitting.

## 1. Introduction

Hydrogen is receiving increasing attention, due to its low carbon footprint and high energy density. The replacement of traditional fossil fuel sources with hydrogen is considered an effective strategy for efficiently solving today’s increasingly serious environmental and energy problems [1]. For large-scale hydrogen production, electrocatalytic water splitting is an efficient and simple method. It is a pollution-free process that does not emit greenhouse gases, unlike other methods such as fossil fuel reforming hydrogen production or methanol/ammonia splitting hydrogen production [2,3]. Hydrogen production from electrocatalytic water can be achieved through various methods, such as photoelectrochemical water oxidation [4,5], alkaline electrocatalytic water splitting [6], electrocatalytic water splitting using proton exchange membranes [7], and solid oxide electrolysis of water for hydrogen production [8]. Alkaline electrocatalytic water splitting is the most mature and widely applicable of these methods [9]. The OER at the anode and the HER at the cathode were the major half-reactions involved in electrocatalytic water splitting. The electrocatalytic splitting of water into H_2_ and O_2_ requires a thermodynamic potential of 1.23 V. However, in practical situations, higher driving voltages are often required to overcome the energy barriers associated with charge transfer and mass transport in the multi-step catalytic process of HER and OER [10,11,12]. The noble metal Pt [13] and Ir [14], etc., demonstrate excellent catalytic performance in electrocatalytic water splitting owing to their appropriate adsorption/desorption free energies for reaction intermediates [15,16]. In spite of this, their high price and rarity prevent them from being widely used as electrocatalysts in HER and OER applications. Therefore, alternative catalysts based on non-noble metals are urgently needed [17,18].

Transition metal compounds’ catalytic materials have the potential to replace noble metals in commercial applications, which can be attributed to their cost-effectiveness and high activity, and the outermost atomic layer electronic structure was controllable, rendering them highly appealing for diverse applications [19]. Ni-, Co-, and Fe-based catalysts, in particular, have demonstrated higher electrocatalytic activity for HER [20,21,22] and OER [23,24,25], making them the focus of interest for many researchers. Bifunctional electrocatalysts can catalyze both OER and HER reactions for water splitting, so the development of electrocatalysts with bifunctional catalytic activity is more valuable for commercial applications. Co_3_O_4_ is a promising candidate catalyst, due to the fact Co_3_O_4_ consists of Co^2+^ and Co^3+^ ions, with three unpaired d electrons on Co^2+^ and all d electrons of Co^3+^ being paired. The bond between cobalt and oxygen exhibits a covalent character in the ion bond dominated Co_3_O_4_ [26]. With its attractive electronic properties, Co_3_O_4_ can serve as a reliable and efficient catalyst in a wide range of electrochemical catalytic reactions [27,28,29]. Due to its rich and controllable surface electronic structure, the electrocatalyst Co_3_O_4_ was also used for water splitting [30,31]. While Co_3_O_4_ exhibits satisfactory OER catalytic activity, it has yet to achieve commercial viability. Additionally, its poor HER activity poses limitations on its overall water splitting performance. Thus, it is imperative to explore practical and feasible approaches to enhance its bifunctional electrocatalytic activity for water decomposition.

The electrocatalytic reaction of water splitting occurs on the surface of a catalyst, and therefore, its surface structure is closely related to its catalytic activity. Various methods exist to modulate the surface properties of a catalyst, such as morphology modulation [32], electronic structure modulation [33], heterojunction engineering [34], and defect engineering [35], etc. Defect engineering is a useful strategy for regulating catalytic performance, since it can modulate the electronic structure of catalyst surfaces and adjust the adsorption/desorption process of reactants and products on the catalyst surface by redistributing charges [36]. Previous studies have demonstrated that as an OER catalyst, surface modifications can alter the catalyst’s electronic structure, reduce reaction intermediates’ (OH*, O*, OOH*) energy barriers and enhance intrinsic catalytic activity [37]. As a HER catalyst, cobalt-based compounds are converted to complexes of cobalt metal and Co^2+^ during the HER process [38]. Cobalt, serving as the active center, contributes significantly to both the adsorption of surface water molecules and protons during HER, and the desorption of HER surface intermediates [39]. Moreover, the replacement of certain anions in cobalt-based compounds has the potential to adjust the electronic configuration of cobalt, thereby lowering the free-energy barrier for H* adsorption and promoting the release of H2 from the active site, thus accelerating the HER. Elements such as C [40], N [41], P [42], and S [43] have been demonstrated to be effective dopants for Co-based catalysts in HER. Introducing cation/anion vacancies onto the surface of a catalyst has been affirmed as an effective strategy for enhancing catalytic activity. Recent studies suggest that dual doping enhances the catalytic activity of catalysts more effectively than single doping [44], and in situ filling of vacancies in the catalyst can lead to significant improvements in catalytic activity, and these improvements are more pronounced than those achieved with a single vacant site [45].

In this study, we attempt to employ a combination of plasma etching and in situ heteroatom filling strategies to etch the surface of Co_3_O_4_ to create abundant oxygen vacancies and fill the oxygen vacancies with N and S heteroatoms simultaneously. Ar plasma etching reduced the size of the Co_3_O_4_ nanosheets, creating a substantial number of defects and exposing more active sites, while the introduction of N and S optimized the surface and structure of the catalyst. The obtained N/S-V_O_-Co_3_O_4_ showed a significant enhancement in the electrocatalytic decomposition activity of water in 1.0 M KOH, especially the HER catalytic activity, compared with the pristine Co_3_O_4_, and N/S-V_O_-Co_3_O_4_ exhibited significant long-term catalytic stability for both HER and OER. Furthermore, the double electrode composed of N/S-V_O_-Co_3_O_4_ exhibited catalytic activity comparable to that of Pt/C and IrO_2_ in an alkaline overall water-splitting simulated electrolytic cell and showed excellent long-term catalytic stability.

## 2. Results and Discussion

**Scheme 1:**
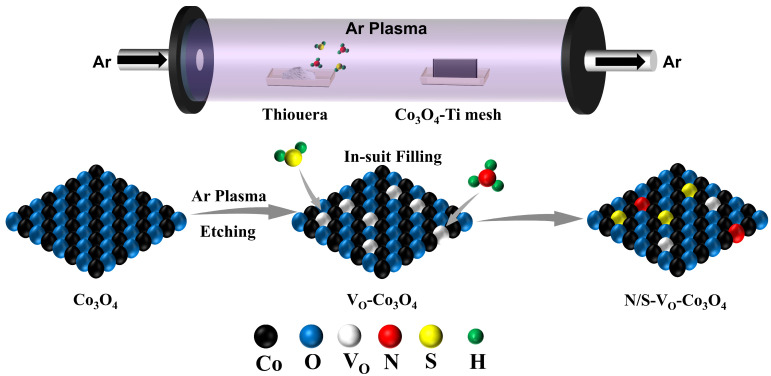
Schematic of Ar plasma-assisted in situ filling of N and S heteroatoms.

The synthesis method for N/S-V_O_-Co_3_O_4_ is illustrated in Scheme 1 (details are given in the Supplementary Materials). Briefly, during the preparation of N/S-V_O_-Co_3_O_4_, oxygen vacancies were etched on the surface of Co_3_O_4_ by using Ar plasma. Simultaneously, NH_3_ and H_2_S generated via the thermal decomposition of thiourea were used as N and S sources to fill the oxygen vacancies in V_O_-Co_3_O_4_.

Initially, we employed the HER and OER characteristics of the catalyst as indicators in a 1.0 M KOH solution. By adjusting the plasma treatment time, we established the optimal preparation conditions. The experimental results showed that the use of Ar plasma assistance for the introduction of N and S heteroatoms could significantly improve the HER and OER properties of the catalyst, as shown in Figure S1. Additionally, with Ar plasma assistance, the catalysts exhibited the highest catalytic activity compared to direct introduction, and surpassed the performance of catalysts with single heteroatoms under equivalent conditions, as depicted in Figure S2. The optimal parameters for the synthesis of N/S-V_O_-Co_3_O_4_ have been determined. Co_3_O_4_, V_O_-Co_3_O_4_, and N/S-V_O_-Co_3_O_4_ were used as the study objects, and the source of catalytic activity of N/S-V_O_-Co_3_O_4_ was explored in the following experiments by combining various characterization and electrochemical measurements.

**Figure 1:**
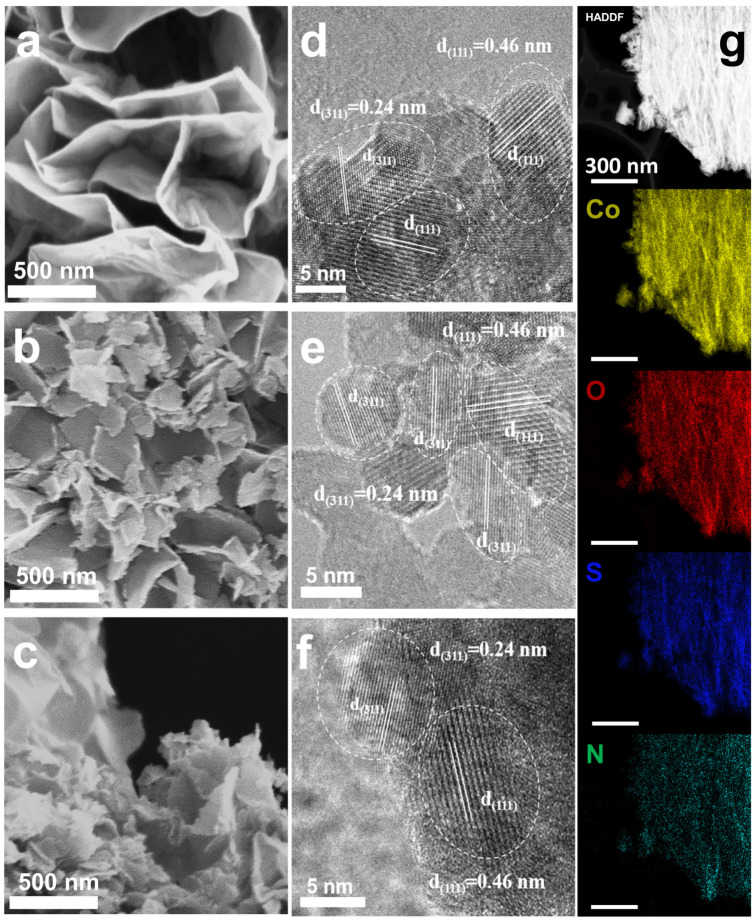
(**a**–**c**) SEM and (**d**–**f**) HRTEM images of Co_3_O_4_, V_O_-Co_3_O_4_, and N/S-V_O_-Co_3_O_4_, respectively. (**g**) HADDF-TEM image and corresponding EDS mappings of N/S-V_O_-Co_3_O_4_.

The catalysts’ morphologies were examined using scanning electron microscopy (SEM); SEM revealed that the morphology of Co_3_O_4_ was observed as nanoclusters that were formed by closely stacked nanosheets, which grew vertically to the substrate and were uniformly distributed, and the surfaces of the pristine Co_3_O_4_ nanosheets were observed to be clean, smooth, and dense, as shown in Figure 1a and Figure S3a. After the plasma-etching process, the surface of the V_O_-Co_3_O_4_ nanosheets became porous, discontinuous, and loosely planar, and the larger nanosheet structure was etched into a smaller nanosheet structure, while bulk phase defects appeared on the nanosheets, as shown in Figure 1b and Figure S3b. Similar to the microscopic morphology of V_O_-Co_3_O_4_, the nanosheets of N/S-V_O_-Co_3_O_4_ became porous, discontinuous, and loosely planar, as shown in Figure 1c and Figure S3c. To further analyze the elemental species and their distribution on the surface of the catalyst, energy-dispersive X-ray spectroscopy (EDS) was used to probe the elemental distribution (mapping) on the surface of the N/S-V_O_-Co_3_O_4_, as seen in Figure S4. The EDS mapping results showed that the elements Co, O, S, and N were evenly distributed on the surface of N/S-V_O_-Co_3_O_4_. Notably, the concentrations of N and S heteroatoms were relatively low, indicating that their presence in the Co_3_O_4_ nanosheets was limited.

To gain further insight into the microscopic morphology and structure of the catalysts, we used transmission electron microscopy (TEM) to analyze their morphology, structure, and elemental distribution. The TEM images revealed that the morphology of Co_3_O_4_ is a nanosheet structure with smooth edges, while the morphology of N/S-V_O_-Co_3_O_4_ bears resemblance to that of V_O_-Co_3_O_4_ following Ar plasma etching. The thickness of the Co_3_O_4_ nanosheet was reduced and the edges of the nanosheet appeared to be chipped, consistent with the SEM results, as shown in Figure S5. High-resolution transmission electron microscopy (HRTEM) was subsequently employed to observe the obtained range of catalysts, as shown in Figure 1d–f. There two diffraction stripes with different lattice spacings were found on Co_3_O_4_, where the lattice stripe with a distance of 0.46 nm corresponds to the (111) crystal plane of Co_3_O_4_, while the lattice stripe of 0.24 nm corresponds to the (311) crystal plane of Co_3_O_4_ (JCPDS 43-1003) [46]. The lattice diffraction stripes on the V_O_-Co_3_O_4_ were the same as those on the Co_3_O_4_, indicating that the Ar plasma etching did not cause a significant structural change for the Co_3_O_4_. Similarly, the lattice diffraction stripes observed on N/S-V_O_-Co_3_O_4_ were the same as those observed on Co_3_O_4_, indicating that the introduction of N and S heteroatoms did not cause significant phase transformation of Co_3_O_4_. The elemental compositions of Co_3_O_4_ and N/S-V_O_-Co_3_O_4_ were obtained via EDS in the High-Angle Annular Dark-Field (HADDF) mode for further analysis of the elemental species and distribution in the nanoclusters, as shown in Figures S6 and S7. The characteristic peaks of N and S elements were observed in the EDS spectrum of N/S-V_O_-Co_3_O_4_; this indicates the successful introduction of N and S. The amount of each element in Co_3_O_4_ and N/S-V_O_-Co_3_O_4_ were shown in Tables S1 and S2, respectively. In N/S-V_O_-Co_3_O_4_, the loading content of N and S elements were 4.31% and 13.29%, respectively, demonstrating that the amount of N and S elements introduced in N/S-V_O_-Co_3_O_4_ was relatively small. Figure 1g shows the EDS mapping, which confirms the homogeneous distribution of the elements Co, O, S, and N on the surface of N/S-V_O_-Co_3_O_4_, compared with Figure S8. It can also be observed that the amounts of N and S elements were relatively small, consistent with the SEM mapping.

**Figure 2:**
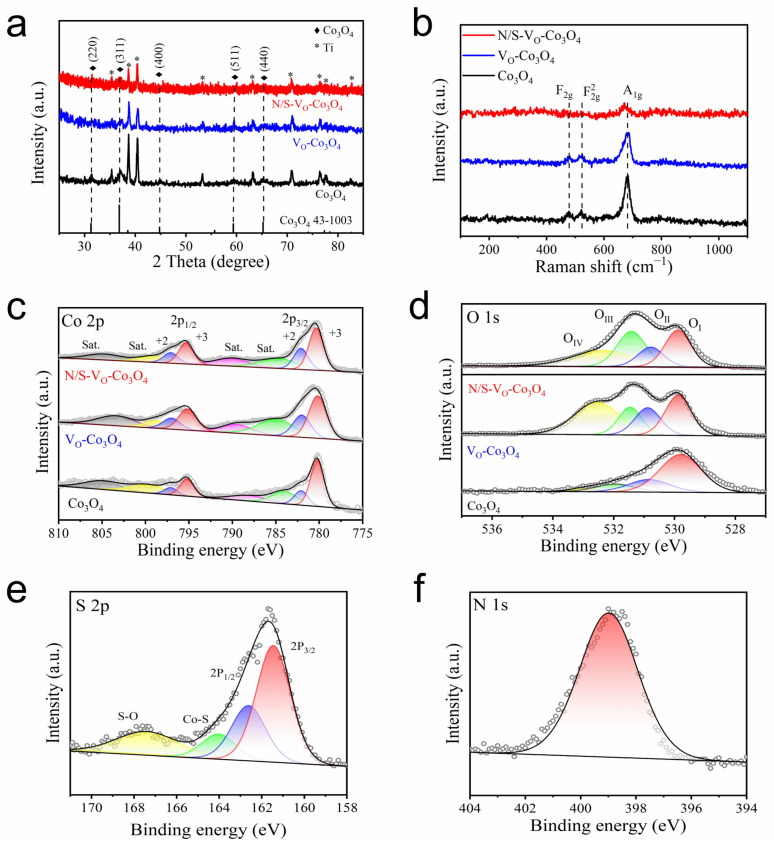
(**a**) XRD spectra. (**b**) Raman spectra. (**c**) Co 2p spectra. (**d**) O 1s spectra of Co_3_O_4_, V_O_-Co_3_O_4_ and N/S-V_O_-Co_3_O_4_. (**e**) S 2p and (**f**) N 1s spectrum of N/S-V_O_-Co_3_O_4_.

The catalysts’ crystal structures were analyzed using X-ray diffraction (XRD). As depicted in Figure 2a, diffraction peaks at 2*θ* = 31.27°, 36.85°, 44.81°, 59.35°, and 65.23° were observed for the obtained range of catalysts, corresponding to the (220), (311), (400), (511), and (440) crystal planes of Co_3_O_4_. (JCPDS 43-1003). This observation suggests that the lattice structure of the catalysts remained largely unchanged despite undergoing Ar plasma etching or introducing N and S heteroatoms. Raman was also utilized to investigate the structural properties of the catalysts, and the Raman spectra of Co_3_O_4_, V_O_-Co_3_O_4_, and N/S-V_O_-Co_3_O_4_ are shown in Figure 2b. There were three obvious peaks observed in the Raman spectra at 475 cm^−1^, 521 cm^−1^, and 683 cm^−1^, corresponding to the $F_{2g}$, $F_{2g}^{2}$, and $A_{1g}$ phonon modes of the Co_3_O_4_ crystals, respectively [47]. There were no other peaks belonging to cobalt sulfate or cobalt nitrate in the Raman spectrum, which indicated that the crystal structure of Co_3_O_4_ remained unchanged after the introduction of N and S heteroatoms. Additionally, a small shift at A_1g_ could be observed, which could be attributed to the different electronegativity [48] of the introduced N and S heteroatoms.

X-ray photoelectron spectroscopy (XPS) was utilized to examine the elemental composition and metal valence states of Co_3_O_4_, V_O_-Co_3_O_4_, and N/S-V_O_-Co_3_O_4_. As shown in the XPS survey spectra of Co_3_O_4_, V_O_-Co_3_O_4_, and N/S-V_O_-Co_3_O_4_, Figure S9. The characteristic peaks of N 1s and S 2p were found only in the survey spectrum of N/S-V_O_-Co_3_O_4_ indicating the successful introducing of N and S heteroatoms. For the Co 2p spectra in Figure 2c, it can be observed that the Co 2p spectra of all catalysts can be decomposed into eight fitting peaks, indicating the presence of different valence states of Co in Co_3_O_4_. The fitting peaks at 780.1 eV and 795.1 eV were attributed to Co^3+^, and the peaks at 782.2 eV and 797.2 eV were attributed to Co^2+^. The remaining four fitting peaks were considered to be satellite peaks of Co 2p [49]. By comparing the areas of the peaks of Co^3+^ and Co^2+^ in the Co 2p spectra of each catalyst, the valence change of Co during the Ar plasma treatment and introduction of N and S heteroatoms can be analyzed. The XPS analysis revealed that the content of Co^2+^ increased following Ar plasma etching. This indicates that the reduction in the valence state of Co was caused by the oxygen vacancies on the catalyst surface induced by Ar plasma etching [50]. Conversely, the Co^2+^: Co^3+^ ratio decreased after introduction of oxygen vacancies with N and S heteroatoms, indicating that the introducing of N and S heteroatoms caused a slight increase in the valence of Co, possibly due to the electronegativity difference compared to O atoms, as seen in Table S3. Figure 2d shows the decomposed O 1s spectra, which exhibits four characteristic peaks (O_I_, O_II_, O_III_, and O_IV_) at the binding energies of 529.9, 530.7, 531.4, and 532.4 eV, respectively. These peaks correspond to the lattice oxygen, oxygen vacancies, surface-adsorbed hydroxyl groups, and surface-adsorbed oxygen-containing species of Co_3_O_4_, respectively [51,52,53]. The oxygen vacancy concentration in the surface of the catalyst can be established by comparing the ratio of O_II_:O_I_ in the O 1s spectrum [54]. Following plasma treatment, the ratio of O_II_:O_I_ in V_O_-Co_3_O_4_ increased, as seen in Table S4, indicating that plasma treatment increased the concentration of the oxygen vacancies of the catalyst. After introduction of N and S heteroatoms, the oxygen vacancy concentration of N/S-V_O_-Co_3_O_4_ decreased; nevertheless, it remained higher than that of the pristine Co_3_O_4_, indicating that N and S heteroatoms maybe in situ filled in the oxygen vacancies, as we reported previously. In the S 2p spectrum of the N/S-V_O_-Co_3_O_4_ in Figure 2e, the S 2p can be decomposed into four fitted peaks and the peaks at 161.5, 162.7, 164.1, and 167.5 eV, which were ascribed to 2P_3/2_, 2P_1/2_, Co-S, and Co-O, respectively [55], indicating the successful introduction of the S atoms and the S atoms bonding with the Co and O atoms on the surface of Co_3_O_4_. In Figure 2f, the N 1s spectrum was ascribed to the bonding of N with metal elements, indicating the successful introduction of N elements [56].

By combining the morphological and structural characterizations of SEM, TEM, XRD, Raman, and XPS, as well as the previous reported experimental design and the characterization results, it is reasonable to speculate that plasma constructs surface oxygen defects while being able to fill the introduced N and S heteroatoms in situ in the oxygen vacancies [56,57,58]. We determined the in situ filling of the oxygen vacancies of Co_3_O_4_ by N and S heteroatoms. Subsequently, we investigated the impact of these modifications on the catalytic performance of the catalyst in the alkaline water electrolysis by means of thorough electrochemical tests.

The electrochemically active specific surface area (ECSA) was utilized to evaluate the variations in intrinsic activity of the range of catalysts via cyclic voltammetry (CV). The CV tests were carried out on Co_3_O_4_, V_O_-Co_3_O_4_, and N/S-V_O_-Co_3_O_4_ with different scan rates (*v*). The linear relationship diagrams, as shown in Figure S10, were drawn based on their respective current density (Δ*J*/2) and *v*. The C*_dl_* of Co_3_O_4_, V_O_-Co_3_O_4_, and N/S-V_O_-Co_3_O_4_ were determined to be 0.43, 0.65, and 1.13 mF cm^−2^, respectively, based on the slopes of the fitted line, and the ECSA value of N/S-V_O_-Co_3_O_4_ was further calculated to be 28.3 cm^2^, which was much larger than that of V_O_-Co_3_O_4_ (16.3 cm^2^) and Co_3_O_4_ (10.8 cm^2^). These outcomes suggest that the introduction of oxygen vacancies effectively improves the ECSA, resulting in a significant increase in the quantity of catalytic reaction sites. Additionally, the number of catalytic sites was further increased by filling the N and S heteroatoms.

**Figure 3:**
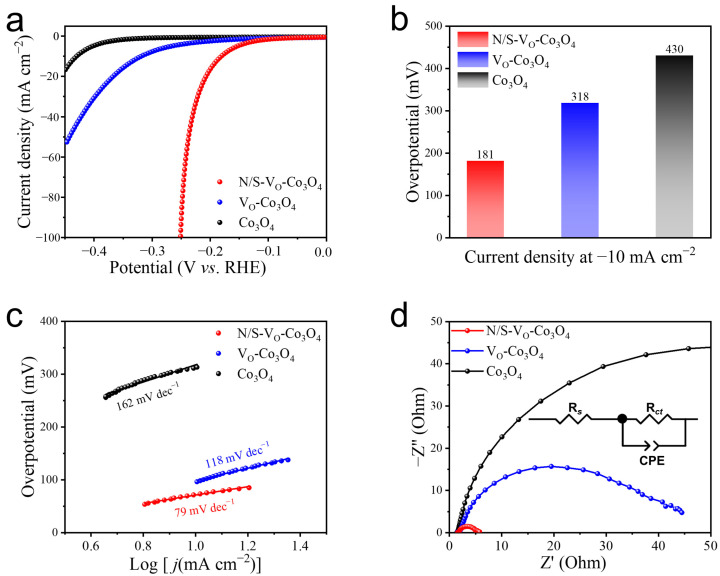
(**a**) HER polarization curves (with 95% iR-compensation), (**b**) corresponding overpotentials at −10 mA cm^−2^, (**c**) Tafel slope, (**d**) Nyquist plots at −1.30 V vs. SCE of the N/S-V_O_-Co_3_O_4_, V_O_-Co_3_O_4_ and Co_3_O_4_.

The HER catalytic activities of Co_3_O_4_, V_O_-Co_3_O_4_, and N/S-V_O_-Co_3_O_4_ were evaluated in a 1.0 M KOH solution. As depicted in Figure 3a,b, the HER catalytic activity of N/S-V_O_-Co_3_O_4_ was greatly improved after Ar plasma etching and in situ filling of N and S heteroatoms; a mere overpotential of 181 mV was required to achieve −10 mA cm^−2^, which is much lower than the overpotentials required for V_O_-Co_3_O_4_ (318 mV) and Co_3_O_4_ (430 mV), respectively. The Tafel slope of each catalyst was shown in Figure 3c, the slope of N/S-V_O_-Co_3_O_4_ was 79 mV dec^−1^, which was much lower than V_O_-Co_3_O_4_ (118 mV dec^−1^) and Co_3_O_4_ (162 mV dec^−1^), while the smaller Tafel slope reflects the faster reaction kinetics of the catalyst during the HER reaction, which is better than most of the Co-based catalysts reported currently, as shown in Table S7 [58,59,60,61,62,63,64,65,66,67]. To further analyze the kinetic processes involved in the HER process, electrochemical impedance spectroscopy (EIS) tests were conducted at −1.3 V vs. SCE. The Charge transfer resistance (R*_ct_*) of each catalyst during the HER reaction was obtained from EIS fitting, with results shown in Table S5. It is generally believed that the smaller the impedance value, the higher the charge transfer efficiency of the corresponding catalyst. Among them, N/S-V_O_-Co_3_O_4_ possesses the smallest electrochemical impedance (R*_ct_* = 4.21 Ω), which is much smaller than that of V_O_-Co_3_O_4_ (R*_ct_* = 35.83 Ω) and Co_3_O_4_ (R*_ct_* = 105.64 Ω), as shown in Figure 3d. The enhancement of the charge transfer rate could be attributed to the incorporation of oxygen vacancies and in situ filling of N and S heteroatoms. The catalytic stability of N/S-V_O_-Co_3_O_4_ for HER was evaluated through 1000 cycles of CV within a range of −0.3 V to 0.1 V vs. RHE at a scan rate of 100 mV s^−1^. The comparison of polarization curves before and after the 1000 cycles CV almost overlap, as shown in Figure S11a. Furthermore, N/S-V_O_-Co_3_O_4_ was subjected to a 12-h durability test at −10 mA cm^−2^, as depicted in Figure S11b. Notably, the potential of the catalyst remained relatively steady throughout the test. Collectively, these results indicate that N/S-V_O_-Co_3_O_4_ demonstrates good catalytic stability during HER.

**Figure 4:**
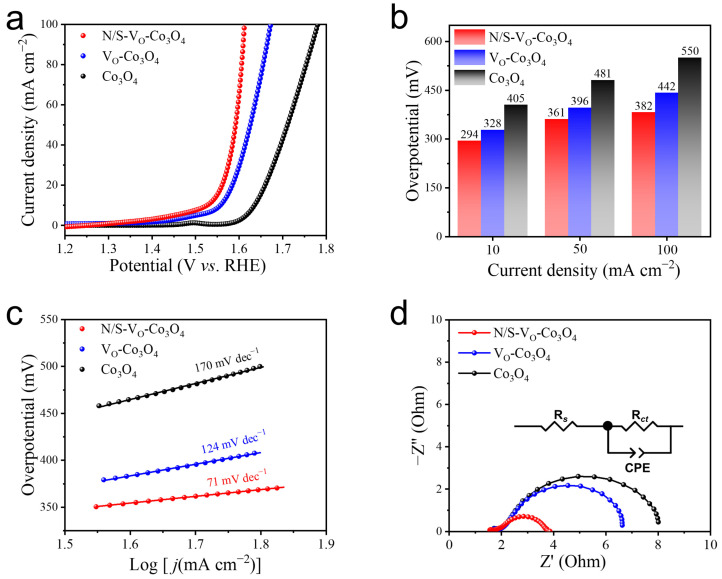
(**a**) OER polarization curves (with 95% iR-compensation), (**b**) Corresponding overpotentials at different current density, (**c**) Tafel slope, (**d**) Nyquist plots at 0.55 V vs. SCE of the N/S-V_O_-Co_3_O_4_, V_O_-Co_3_O_4_ and Co_3_O_4_.

The OER catalytic activity of Co_3_O_4_, V_O_-Co_3_O_4_, and N/S-V_O_-Co_3_O_4_ was also evaluated and compared. As depicted in Figure 4a,b, the OER activity of N/S-V_O_-Co_3_O_4_ required an overpotential of 294 mV to reach 10 mA cm^−2^, which was lower than that of V_O_-Co_3_O_4_ (328 mV) and Co_3_O_4_ (405 mV). Additionally, the Tafel slope of each catalyst was shown in Figure 4c; the Tafel slope of N/S-V_O_-Co_3_O_4_ (71 mV dec^−1^) was lower than that of V_O_-Co_3_O_4_ (124 mV dec^−1^) and Co_3_O_4_ (170 mV dec^−1^), reflecting the faster OER reaction kinetics of the N/S-V_O_-Co_3_O_4_ catalyst due to the smaller Tafel slope, indicating it is better than most of the Co-based catalysts reported currently, as seen in Table S8 [56,60,68,69,70,71,72,73,74,75,76]. EIS was also performed to further analyze the kinetic processes in the OER process, at 0.55 V vs. SCE. As shown in Figure 4d, N/S-V_O_-Co_3_O_4_ has the smallest electrochemical impedance (R*_ct_* = 2.12 Ω), which is smaller than that of V_O_-Co_3_O_4_ (R*_ct_* = 4.68 Ω) and Co_3_O_4_ (R*_ct_* = 6.10 Ω); the EIS fitting results are shown in Table S6. This indicates that the OER charge transfer rate and kinetics of N/S-V_O_-Co_3_O_4_ were also enhanced.

Finally, the OER stability of N/S-V_O_-Co_3_O_4_ was also tested using 1000 cycles of CV in the range of 1.2 to 1.6 V vs. SCE. The OER polarization curves before and after 1000 cycles of CV are shown in Figure S12a, and the LSV curves before and after 1000 cycles of CV almost overlap. Additionally, N/S-V_O_-Co_3_O_4_ was subjected to a 12-h durability test at 10 mA cm^−2^, as depicted in Figure S12b; the potential of the catalyst remained relatively steady throughout the test. In summary, N/S-V_O_-Co_3_O_4_ also demonstrated good OER catalytic durability.

The prepared catalysts were compared with benchmark noble metal-based catalysts Pt/C and IrO_2_, as illustrated in Figure S13. Despite requiring a higher overpotential than Pt/C to achieve −10 mA cm^−2^ in HER, N/S-V_O_-Co_3_O_4_ exhibited a smaller Tafel slope, indicating faster HER kinetics than Pt/C. Regarding OER, N/S-V_O_-Co_3_O_4_ exhibited both a lower overpotential required at 10 mA cm^−2^ and a smaller Tafel slope than IrO_2_, suggesting faster OER kinetics for N/S-V_O_-Co_3_O_4_ than for IrO_2_.

**Figure 5:**
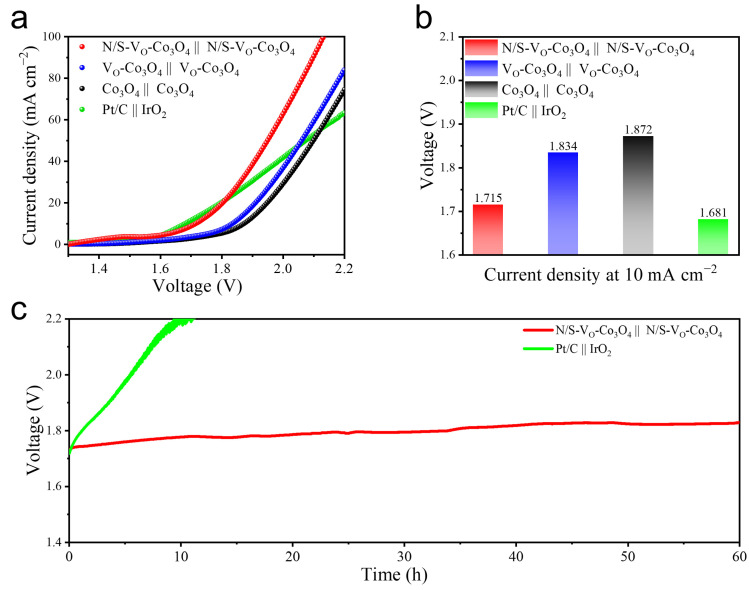
(**a**) Polarization curves and (**b**) corresponding voltages at 10 mA cm^−2^ of the N/S-V_O_-Co_3_O_4_ || N/S-V_O_-Co_3_O_4_, V_O_-Co_3_O_4_ || V_O_-Co_3_O_4_, Co_3_O_4_ || Co_3_O_4_ and Pt/C || IrO_2_. (**c**) Chronopotentiometry curves of the N/S-V_O_-Co_3_O_4_ || N/S-V_O_-Co_3_O_4_ and Pt/C || IrO_2_ at 10 mA cm^−2^. All the stability date without iR compensation.

Benefiting from the gratifying bifunctional activity of N/S-V_O_-Co_3_O_4_ in alkaline electrolytes, N/S-V_O_-Co_3_O_4_ exhibits catalytic activity comparable to that of Pt/C || IrO_2_ in an alkaline overall water-splitting simulated electrolytic cell. As shown in Figure 5a,b, the required voltage of the N/S-V_O_-Co_3_O_4_ || N/S-V_O_-Co_3_O_4_ dual-electrode at the current density of 10 mA cm^−2^ was only 1.715 V, which is much lower than that of the dual-electrode electrolytic cell consisting of V_O_-Co_3_O_4_ || V_O_-Co_3_O_4_ (1.834 V) and Co_3_O_4_ || Co_3_O_4_ (1.872 V), and comparable to Pt/C || IrO_2_ (1.681 V). Moreover, the N/S-V_O_-Co_3_O_4_ || N/S-V_O_-Co_3_O_4_ dual-electrode exhibited favorable electrocatalytic stability in 1.0 M KOH, as shown in Figure 5c, with no significant voltage increase at a constant current density of 10 mA cm^−2^ for 60 h, showing that it outperformed that of Pt/C||IrO_2_.

**Figure 6:**
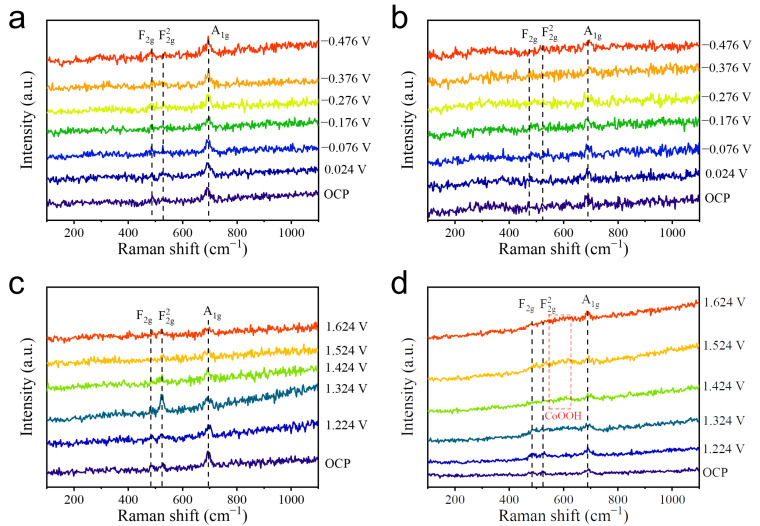
In situ Raman spectra of Co_3_O_4_ and N/S-V_O_-Co_3_O_4_ during HER and OER. (**a**) Co_3_O_4_ in HER. (**b**) N/S-V_O_-Co_3_O_4_ in HER. (**c**) Co_3_O_4_ in OER. (**d**) N/S-V_O_-Co_3_O_4_ in OER.

To gain further insight into the mechanism underlying the bifunctional catalytic activity of N/S-V_O_-Co_3_O_4_ in electrolytic water splitting, we conducted in situ Raman analysis in 1.0 M KOH to dynamically monitor the HER and OER processes of N/S-V_O_-Co_3_O_4_. For comparison, Co_3_O_4_ was also measured under the same conditions. As illustrated in Figure 6a,b, the Raman spectra of Co_3_O_4_ and N/S-V_O_-Co_3_O_4_ did not exhibit significant changes after the applied potential during HER, consistent with the ex situ Raman results in air presented in Figure 2b. This observation indicates that the lattice structure of Co_3_O_4_ did not undergo significant changes during HER. Therefore, it is reasonable to hypothesize that the in situ filled N and S heteroatoms served as moderators on the adsorption/desorption of the reaction intermediates during HER, which enhanced the HER activity of N/S-V_O_-Co_3_O_4_ [41,43]. In contrast, during OER, a new characteristic peak becomes observable in the Raman spectra of N/S-V_O_-Co_3_O_4_, ranging from 550 to 620 cm^−1^, once the applied potential reached 1.424 V vs. RHE, as illustrated in Figure 6d. This peak is indicative of the production of CoOOH [77], while this phenomenon was not observed in Co_3_O_4_, as shown in Figure 6c. Previous reports suggest that the high-valent Co^4+^ in CoOOH is the truly active phase of Co-based catalysts during OER in alkaline electrolytes; it can be inferred that the transition from Co_3_O_4_ to CoOOH is facilitated by the in situ filling of oxygen vacancies with N and S heteroatoms, leading to a lower overpotential required for OER.

Ex situ characterization was also performed on N/S-V_O_-Co_3_O_4_ to analyze its structural changes after the catalytic reaction. Figure S14a shows the XRD results, which indicate that the structure of N/S-V_O_-Co_3_O_4_ did not undergo significant changes before and after the electrocatalytic water splitting. The spinel structure of Co_3_O_4_ was still maintained. On the other hand, a slight shift can be observed in the Co 2p spectrum of N/S-V_O_-Co_3_O_4_ after OER, as shown in Figure S14b, indicating the production of more high-valent Co. This shift was not observed after HER. Figure S14c illustrates the change in the S 2p spectrum of N/S-V_O_-Co_3_O_4_ after the catalytic reaction. When compared with the initial N/S-V_O_-Co_3_O_4_, it was observed that the S species did not undergo significant changes after HER. However, after OER, all S species could be observed to shift to the S–O bonded form. This shift is related to the in situ reconfiguration of N/S-V_O_-Co_3_O_4_ during the OER process. Figure S14d demonstrated that the elemental species of N composition did not undergo significant changes before and after HER and OER. The N species exists in the form of N bonded to metal elements. In summary, it can be concluded that the lattice structure of N/S-V_O_-Co_3_O_4_ remains stable during the HER reaction, and the elemental species of N and S do not undergo significant changes. However, during the OER process, N/S-V_O_-Co_3_O_4_ undergoes a structural transformation, leading to the production of more CoOOH. Furthermore, the S element is further oxidized to produce ${SO}_{4}^{2-}$ during the structural transformation phase.

## 3. Conclusions

In summary, we successfully etched the spinel-structured Co_3_O_4_ nanosheets via Ar plasma to create oxygen vacancy-rich nanosheets (V_O_-Co_3_O_4_) and subsequently filled the oxygen vacancies with N and S heteroatoms to form N/S-V_O_-Co_3_O_4_. SEM and TEM characterization revealed that the Ar plasma etching generated numerous porous defects on the surface of the Co_3_O_4_ nanosheet, which increased the specific surface area of the catalyst and promoted adsorption and desorption of reactants and products in electrocatalytic water splitting. Furthermore, XRD and Raman analysis demonstrated that the in situ filling of N and S atoms did not significantly alter the crystal structure of Co_3_O_4_, indicating the preservation of the crystal structure of Co_3_O_4_ in the obtained catalysts. However, XPS analysis confirmed that the surface electronic structure of Co and O in the catalysts underwent a transformation. This transformation resulted in a significant improvement in the electrocatalytic activity of N/S-V_O_-Co_3_O_4_ for water splitting in a 1.0 M KOH electrolyte. The HER and OER performances of N/S-V_O_-Co_3_O_4_ were greatly enhanced compared to pristine Co_3_O_4_. It only required 181 mV (for HER) and 294 mV (for OER) overpotentials to reach a current density of 10 mA cm^−2^. These values were much lower than the overpotentials of 430 mV (for HER) and 405 mV (for OER) required for pristine Co_3_O_4_. Additionally, N/S-V_O_-Co_3_O_4_ exhibits good catalytic stability. Benefiting from the favorable bifunctional HER and OER electrocatalytic activity, the dual-electrode N/S-V_O_-Co_3_O_4_ || N/S-V_O_-Co_3_O_4_ required 1.715 V to reach the current density of 10 mA cm^−2^, in an alkaline overall water-splitting simulated electrolytic cell, which was comparable to a dual-electrode consisting of Pt/C || IrO_2_. Moreover, N/S-V_O_-Co_3_O_4_ || N/S-V_O_-Co_3_O_4_ exhibited good catalytic stability in long-term overall water splitting compared to Pt/C || IrO_2_. The in situ Raman analysis further revealed that the in situ filled N and S heteroatoms played a moderating role in the adsorption/desorption of reaction intermediates during HER and facilitated the transition from Co_3_O_4_ to CoOOH during OER. This study offers a new approach for the development of effective dual-heteroatom-introduced electrocatalysts for alkaline electrocatalytic overall water splitting.

## Data Availability

The data presented in this study are available on request from the corresponding author.

## Supplementary Materials

The following supporting information can be downloaded at: https://www.mdpi.com/article/10.3390/molecules28104134/s1, Preparation, measurement and characterization details, Figure S1–S14, Table S1–S8. Figure S1. (a) HER and (b) OER polarization curves of N/S-V_O_-Co_3_O_4_ with different plasma treatment times in 1.0 M KOH. Figure S2. (a, c) HER and (b, d) OER polarization curves of catalysis compared with different treatment in 1.0 M KOH. Figure S3. SEM images of (a) Co_3_O_4_, (b) V_O_-Co_3_O_4_, (c) N/S-V_O_-Co_3_O_4_. Figure S4. SEM image and corresponding EDS mappings of N/S-V_O_-Co_3_O_4_. Figure S5. TEM images of (a) Co_3_O_4_, (b) V_O_-Co_3_O_4_, (c) N/S-V_O_-Co_3_O_4_. Figure S6. EDS Spectrum of Co_3_O_4_. Figure S7. EDS Spectrum of N/S-V_O_-Co_3_O_4_. Figure S8. HADDF-TEM image and corresponding EDS mappings of Co_3_O_4_. Figure S9. XPS survey of Co_3_O_4_, V_O_-Co_3_O_4_ and N/S-V_O_-Co_3_O_4_. Figure S10. Cyclic voltammogram curves of (a) Co_3_O_4_, (b) V_O_-Co_3_O_4_, (c) N/S-V_O_-Co_3_O_4_. (d) Corresponding C*_dl_* value of Co_3_O_4_, V_O_-Co_3_O_4_ and N/S-V_O_-Co_3_O_4_, respectively. Figure S11. (a) HER polarization curves of N/S-V_O_-Co_3_O_4_ initial and after 1000 cycles CV. (b) Chronopotentiometry curve of N/S-V_O_-Co_3_O_4_ at −10 mA cm^−2^. Figure S12. (a) OER polarization curves of N/S-V_O_-Co_3_O_4_ initial and after 1000 cycles CV. (b) Chronopotentiometry curve of N/S-V_O_-Co_3_O_4_ at 10 mA cm^−2^. Figure S13. The (a) HER and (b) OER polarization curves of different catalysts in 1.0 M KOH. (c) Tafel slope of N/S-V_O_-Co_3_O_4_ compared with Pt/C, during HER. (d) Tafel slope of N/S-V_O_-Co_3_O_4_ compared with IrO_2_, during OER. Figure S14. (a) XRD, (b) Co 2p, (c) S 2p and (d) N 1s spectra of N/S-V_O_-Co_3_O_4_ before and after HER/OER. Table S1. Elemental ratios of Co_3_O_4_ from EDS spectrum. Table S2. Elemental ratios of N/S-V_O_-Co_3_O_4_ from EDS spectrum. Table S3. The relative ratio of Co^2+^ and Co^3+^ in N/S-V_O_-Co_3_O_4_, V_O_-Co_3_O_4_ and Co_3_O_4_ by Co 2p spectra fitting. Table S4. The relative ratio of oxygen vacancies in N/S-V_O_-Co_3_O_4_, V_O_-Co_3_O_4_ and Co_3_O_4_ by Co 2p spectra fitting. Table S5. Electrochemical impedance spectroscopy fitting results for N/S-V_O_-Co_3_O_4_, V_O_-Co_3_O_4_, Co_3_O_4_ during HER at −1.30 V vs. SCE. Table S6. Electrochemical impedance spectroscopy fitting results for N/S-V_O_-Co_3_O_4_, V_O_-Co_3_O_4_, Co_3_O_4_ during OER at 0.55 V vs. SCE. Table S7. Comparison of HER activity for N/S-V_O_-Co_3_O_4_ and recently reported Co-based catalysts. Table S8. Comparison of OER activity for N/S-V_O_-Co_3_O_4_ and recently reported Co-based catalysts.
