# Supplementary material for: In Situ Filling of the Oxygen Vacancies with Dual Heteroatoms in Co3O4 for Efficient Overall Water Splitting
Source: Molecules. 2023 May 16;28(10):4134. doi: 10.3390/molecules28104134 (PMC10222571; doi:10.3390/molecules28104134)
Supplement: Supplementary file 1 [file molecules-28-04134-s001.zip › molecules-2383169-supplementary.pdf]

Supplementary Materials

In-siut Filling the Oxygen Vacancies with Dual Heteroatoms in Co_3O_4 for Efficient Overall Water Splitting

Wei Duan, Shixing Han, Zhonghai Fang, Zhaohui Xiao, Shiwei Lin

State Key Laboratory of Marine Resource Utilization in South China Sea, School of Materials Science and Engineering, Hainan University, No.58 Renmin Road, Haikou 570228, China

1. Reagents

Cobalt nitrate hexahydrate ($Co(NO_3)_2{\cdot}6H_2O$), thiourea (CH_4N_2S), urea (CH_4N_2O), were purchased from Sigma-Aldrich, Potassium hydroxide (KOH) was purchased from Macklin Reagent, sulfur powder(S) was purchased from Guangzhou Chemical Reagent Factory, nitric acid (HNO_3), hydrofluoric acid (HF) and ethanol (C_2H_5OH) were purchased from Sinopharm Chemical Reagent Co. Ltd. Nafion solution (5 wt% solution, DuPont) was obtained from Sigma Aldrich, commercial Pt/C (20 wt%) was purchased from Hesen Co., Ltd. Commercial IrO_2 was purchased from Macklin Reagent. All chemicals were used without further purification. Deionized Mini-Q water was employed as solvent.

2. Materials and Methods Preparation of catalysts

2.1 Preparation of N/S-V_O-Co_3O_4

A 100 mM aqueous $Co(NO_3)_2$ solution was prepared as the electrolyte, and the $Co(OH)_2$ precursors were electrodeposited at -1.0 V *vs.* Ag/AgCl for 600 s, where titanium mesh, KCl saturated Ag/AgCl electrode and graphite rod as the working electrode (WE), reference electrode (RE) and counter electrode (CE), respectively, the obtained $Co(OH)_2$ wetting with deionized water for 3 times and then dried. The obtained $Co(OH)_2$ was heated to 350 °C in a muffle furnace with a heating rate of 5 °C min^{-1} and then held for 2 h to obtain Co_3O_4 nanosheets.

Co_3O_4 was placed in the rear part of the plasma reactor, 0.76 g of thiourea (CH_4N_2S) as the nitrogen and sulfur source and placed in the front part of the plasma reactor, and Argon was used as the plasma source and the carrier for transporting N and S precursors. The N/S-V_O-Co_3O_4 was obtained by rapidly heating CH_4N_2S to 200 °C at a heating rate of 10 °C min^{-1} and then treating Co_3O_4 with Ar plasma (13.56 MHz RF) for different treatment time (10, 20, 30 and 40 min) at 10 Pa and 50 W, where the sample treated for 30 min was used for further studies.

2.2 Preparation of Comparative Catalysts

Preparation of V_O-Co_3O_4: Use the same preparation method as N/S-V_O-Co_3O_4 without thiourea.

Preparation of N/S-Co_3O_4: Use the same preparation method as N/S-V_O-Co_3O_4, without Ar plasma treatment.

Preparation of N-V_O-Co_3O_4: Use the same preparation method as N/S-V_O-Co_3O_4, replace the thiourea with urea.

Preparation of S-V_O-Co_3O_4: Use the same preparation method as N/S-V_O-Co_3O_4, replace the thiourea with sulfur powder.

Preparation of Pt/C electrodes：5 mg commercial Pt/C catalyst was dispersed in 475 μL isopropanol, then 25 μL Nafion solution was added and sonicated for 30 min to uniformly distribute the catalyst in the solution, and 100 μL of the mixture was added dropwise on the titanium mesh (1 cm × 2 cm) with an effective electrode area of 1.0 cm^2, and natural air-drying was applied, the loading of Pt/C was 1.0 mg cm^{-2}.

Preparation of IrO_2 electrodes：The preparation is similar to the Pt/C electrode, but replacing Pt/C with IrO_2，the loading of IrO_2 was 1.0 mg cm^{-2}.

3. Characterization methods

Scanning electron microscopy (SEM, JEOL JSM-7800F) was used to characterize the microscopic morphology and elemental distribution information of the samples, working at a voltage of 10 kV. Transmission electron microscopy (TEM, Thermo Scientific Talos F200X G2) was used to characterize the microscopic morphology, lattice structure and elemental distribution of the samples, working at a voltage of 100 KV. X-ray diffractometer (XRD, DX-2700BH) was used to characterize the structure of the samples (Cu Kα, 2.4 kW), test angle was 5° to 80°, scanning rate was 10° min^{-1}. Laser Raman spectroscopy (Raman, inVia Reflex) was used to analyze the structural information of the samples with a laser wavelength of 514 nm. The X-ray photoelectron spectrometer (XPS, Thermo Scientific ESCALAB 250Xi) was used to test the surface elemental chemical environment and valence information of the samples, the X-ray source was Al Kα with an energy of 1486.6 eV. The binding energies of the XPS spectra included in this work have been corrected by the binding energy of C 1s (284.8 eV).

4. Electrochemical measurements

All electrochemical measurements were performed on IviumStat workstation. During the electrochemical tests, a series of obtained catalysts were used as working electrodes (WE) for the three-electrode system, Saturated Calomel Electrode (SCE) and Graphite Rods as the reference electrodes (RE) and counter electrodes (CE), respectively, and 1.0 M aqueous KOH solution was used as the electrolyte. All polarization curves were tested using a scanning rate of 5 mV s^{-1}, and all polarization curves were tested without iR compensation unless otherwise stated.

The electrode potential is converted to the reversible hydrogen electrode potential (RHE) by the Nernst equation, with the Equation (1):

$$E\ (vs.\ RHE) = E\ (vs.\ SCE) + 0.059 \times pH + 0.241\ V \quad (1)$$

Electrochemical impedance spectroscopy (EIS) tests were performed in the frequency range of 100 kHz to 0.01 Hz with an AC amplitude of 10 mV. EIS curves were tested at potentials of 0.5 V (*vs.* SCE) for the OER and -1.3 V (*vs.* SCE) for the HER. The Tafel slope is calculated as Equation (2):

$$\eta = a + b\ Log|\ j\ | \quad (2)$$

where η is the overpotential, a is the Tafel constant, and b is the Tafel slope.

The electrochemically active specific surface area (ECSA) was estimated from the double-layer capacitance value (C_{dl}), which was tested and calculated by cyclic voltammetry in the non-Faraday region. The selected potential sweep range was -0.7 to -0.80 V *vs.* SCE with sweep rates of 2, 4, 6, 8, and 10 mV s^{-1}. The ECSA was calculated as Equation (3):

$$ECSA = C_{dl}/C_s \times A \quad (3)$$

where C_s is the specific capacitance with a value of about 0.04 mF cm^{-2} and A is the reaction area of the electrode (1.0 cm^2).

The stability test was divided into two parts, one was to test the polarization curves before and after 1000 cycles CV respectively, and the second test method was a Chrono-potentiometry test at a current density of 10 mA cm^{-2}.

5. Supplementary Figures and Table

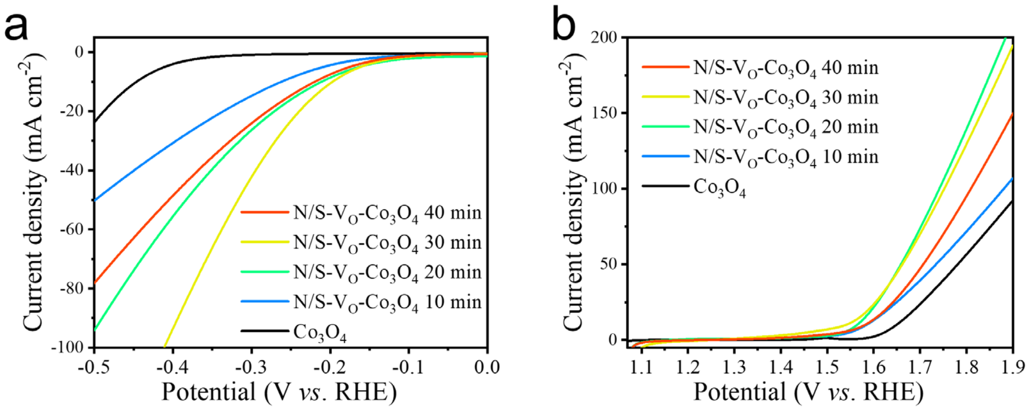

Figure S1. (**a**) HER and (**b**) OER polarization curves of N/S-V_O-Co_3O_4 with different plasma treatment times in 1.0 M KOH.

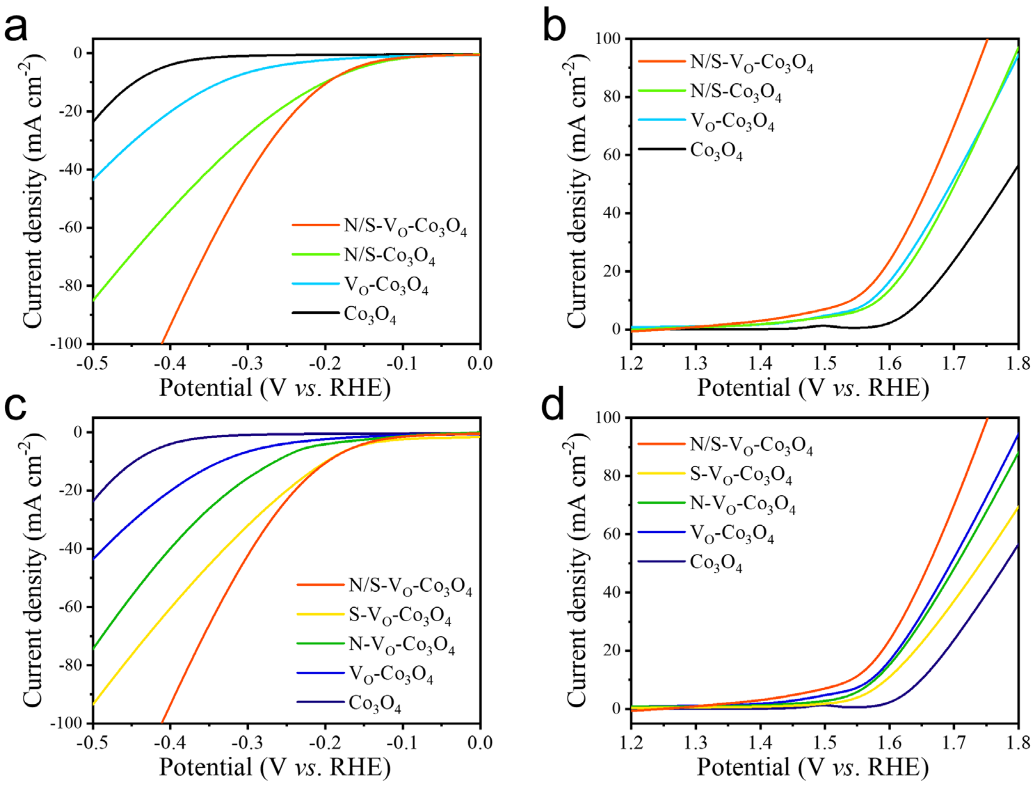

Figure S2. (**a, c**) HER and **(b, d**) OER polarization curves of catalysis compared with different treatment in 1.0 M KOH.

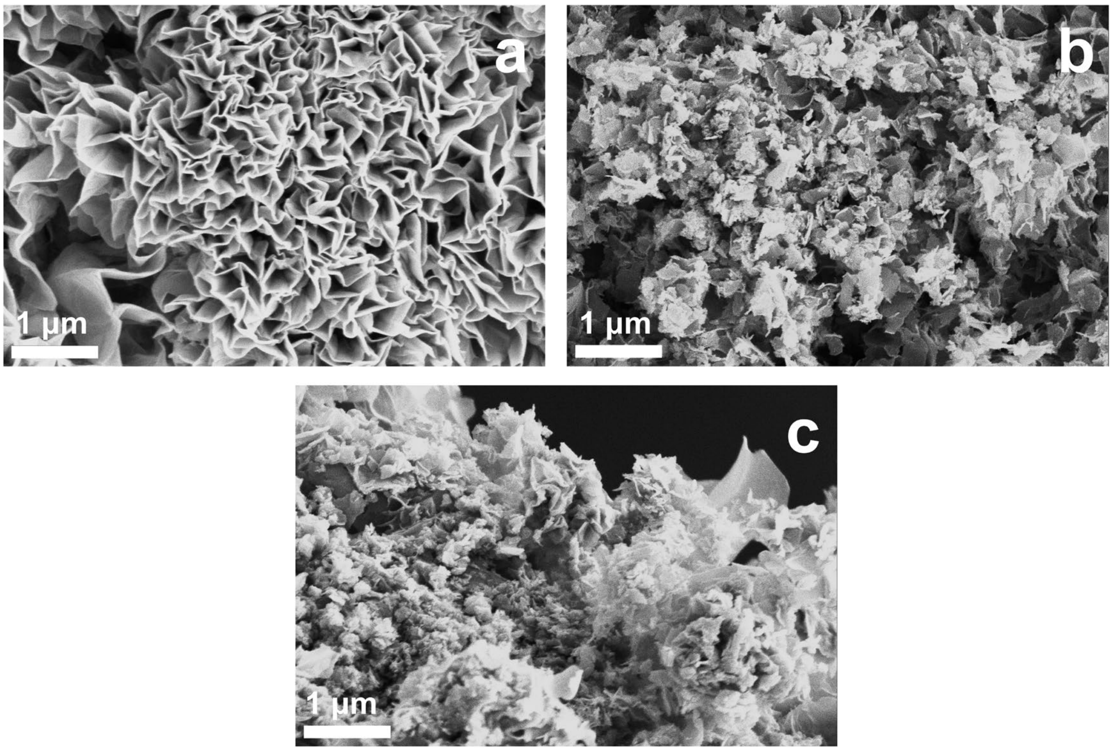

Figure S3. SEM images of (**a**) Co_3O_4, (**b**) V_O-Co_3O_4, (**c**) N/S-V_O-Co_3O_4.

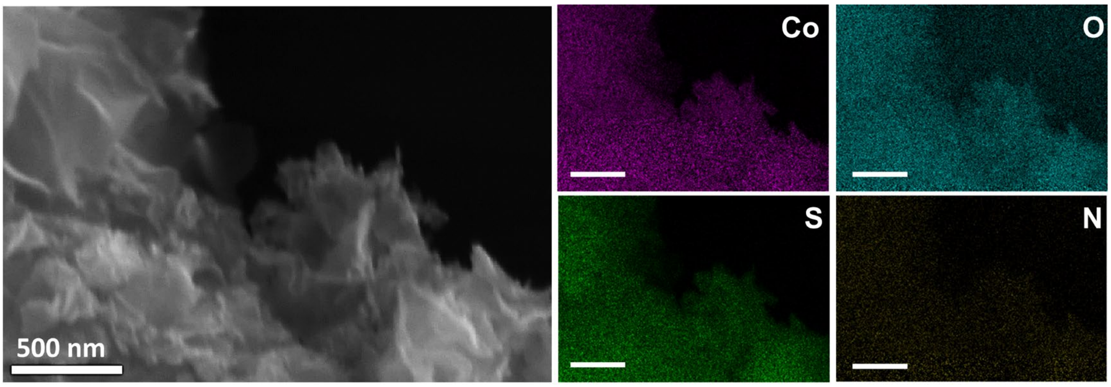

Figure S4. SEM image and corresponding EDS mappings of N/S-V_O-Co_3O_4.

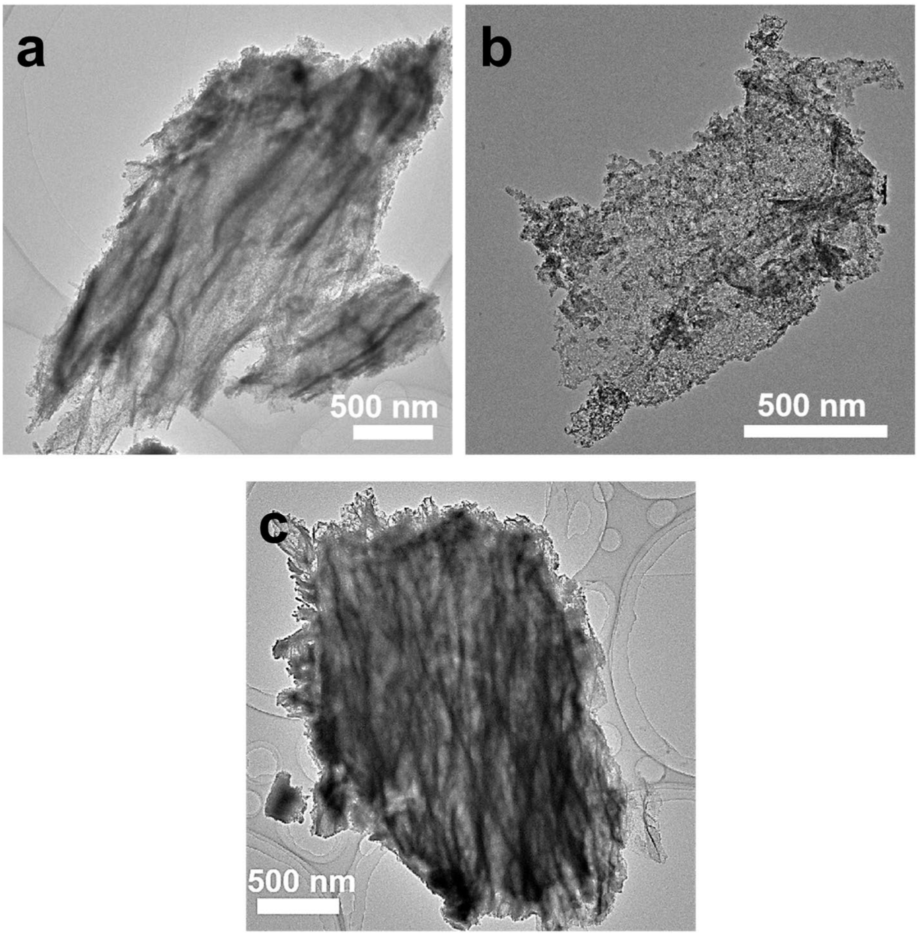

Figure S5. TEM images of (**a**) Co_3O_4, (**b**) V_O-Co_3O_4, (**c**) N/S-V_O-Co_3O_4.

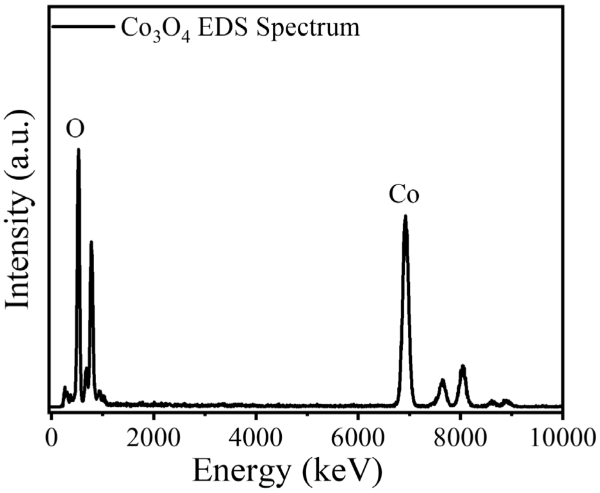

Figure S6. EDS Spectrum of Co_3O_4.

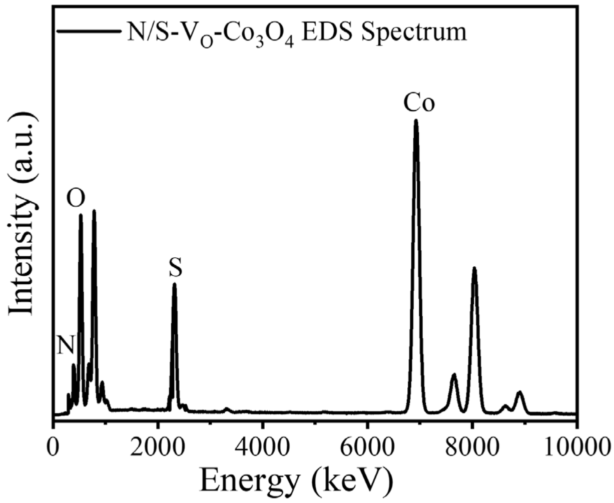

Figure S7. EDS Spectrum of N/S-V_O-Co_3O_4.

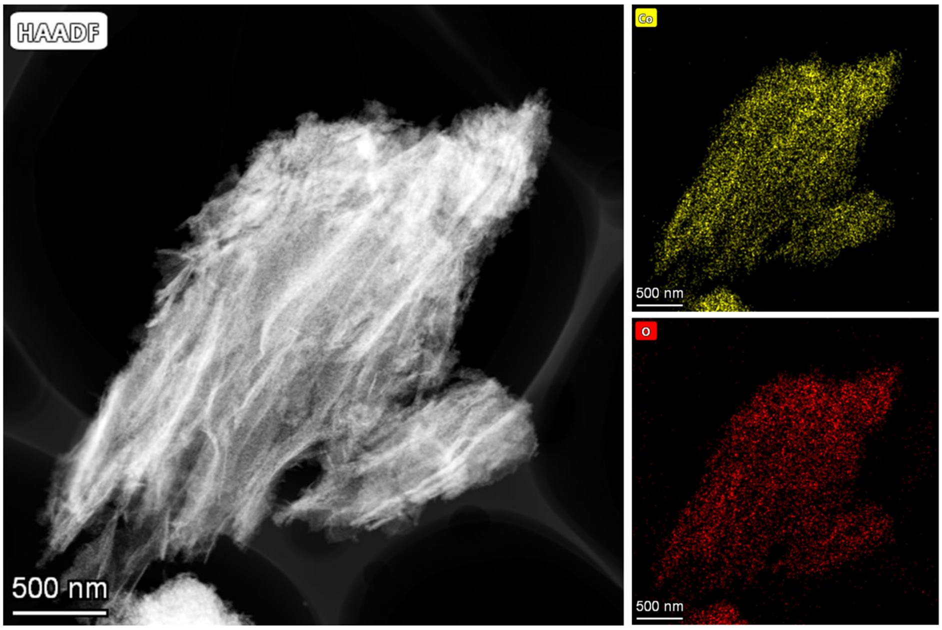

Figure S8. HADDF-TEM image and corresponding EDS mappings of Co_3O_4

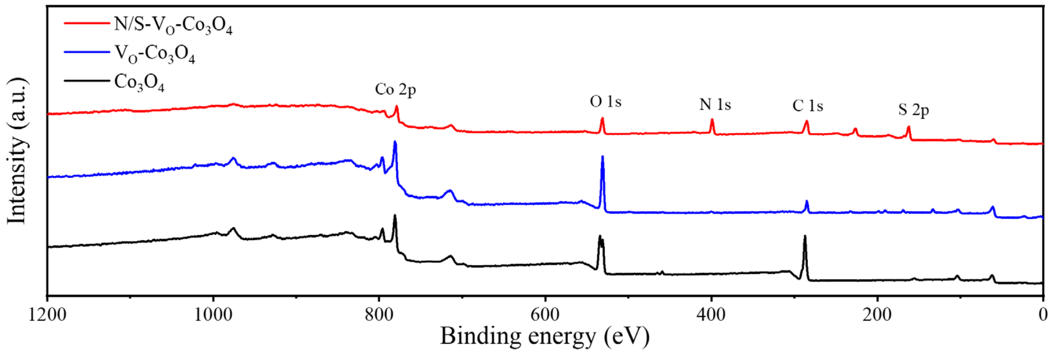

Figure S9. XPS survey of Co_3O_4, V_O-Co_3O_4 and N/S-V_O-Co_3O_4.

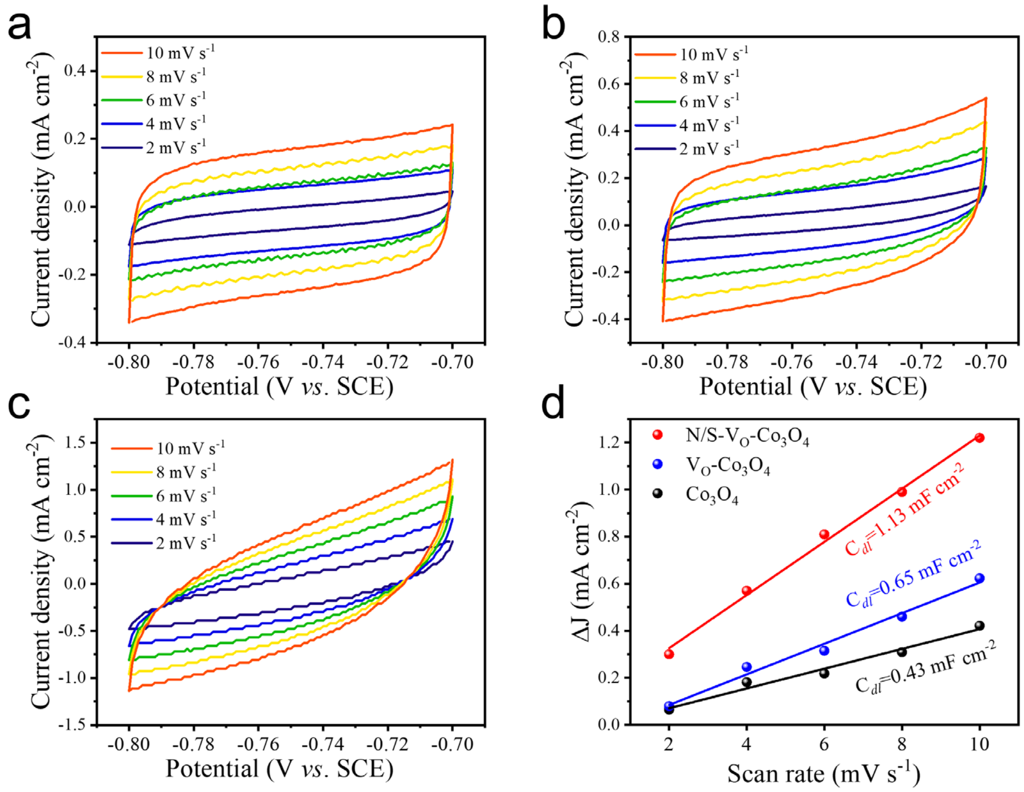

Figure S10. Cyclic voltammogram curves of (**a**) Co_3O_4, (**b**) V_O-Co_3O_4, (**c**) N/S-V_O-Co_3O_4. (**d**) Corresponding C_{dl} value of Co_3O_4, V_O-Co_3O_4 and N/S-V_O-Co_3O_4, respectively.

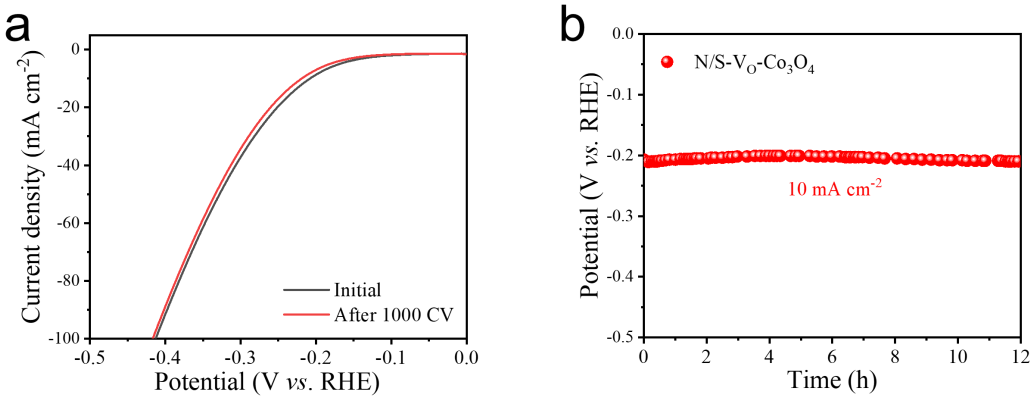

Figure S11. (**a**) HER polarization curves of N/S-V_O-Co_3O_4 initial and after 1000 cycles CV. (**b**) Chronopotentiometry curve of N/S-V_O-Co_3O_4 at -10 mA cm^{-2}.

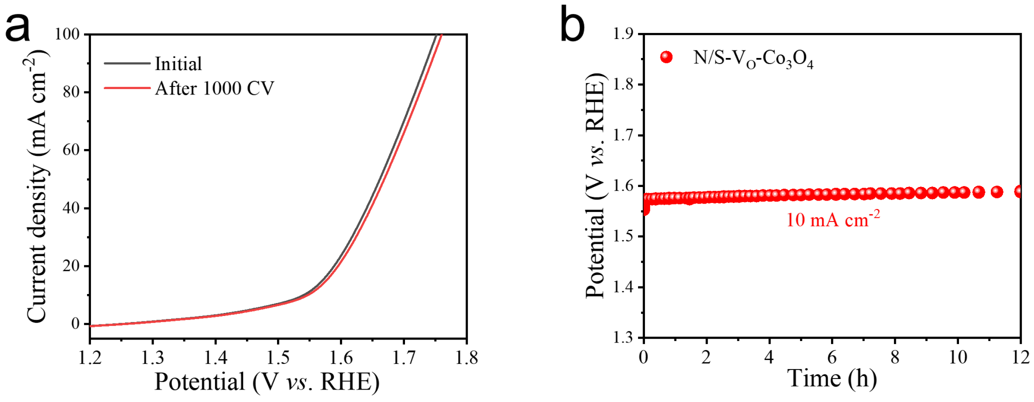

Figure S12. (**a**) OER polarization curves of N/S-V_O-Co_3O_4 initial and after 1000 cycles CV. (**b**) Chronopotentiometry curve of N/S-V_O-Co_3O_4 at 10 mA cm^{-2}.

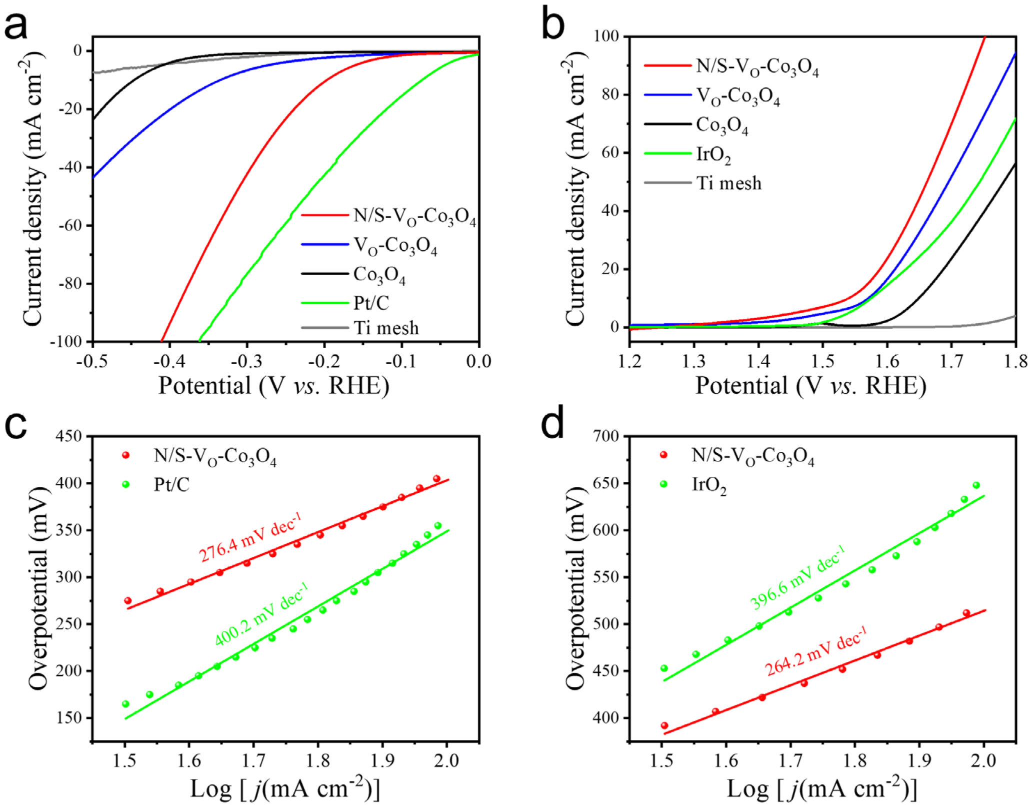

Figure S13. The (**a**) HER and (**b**) OER polarization curves of different catalysts in 1.0 M KOH. (**c**) Tafel slope of N/S-V_O-Co_3O_4 compared with Pt/C, during HER. (**d**) Tafel slope of N/S-V_O-Co_3O_4 compared with IrO_2, during OER.

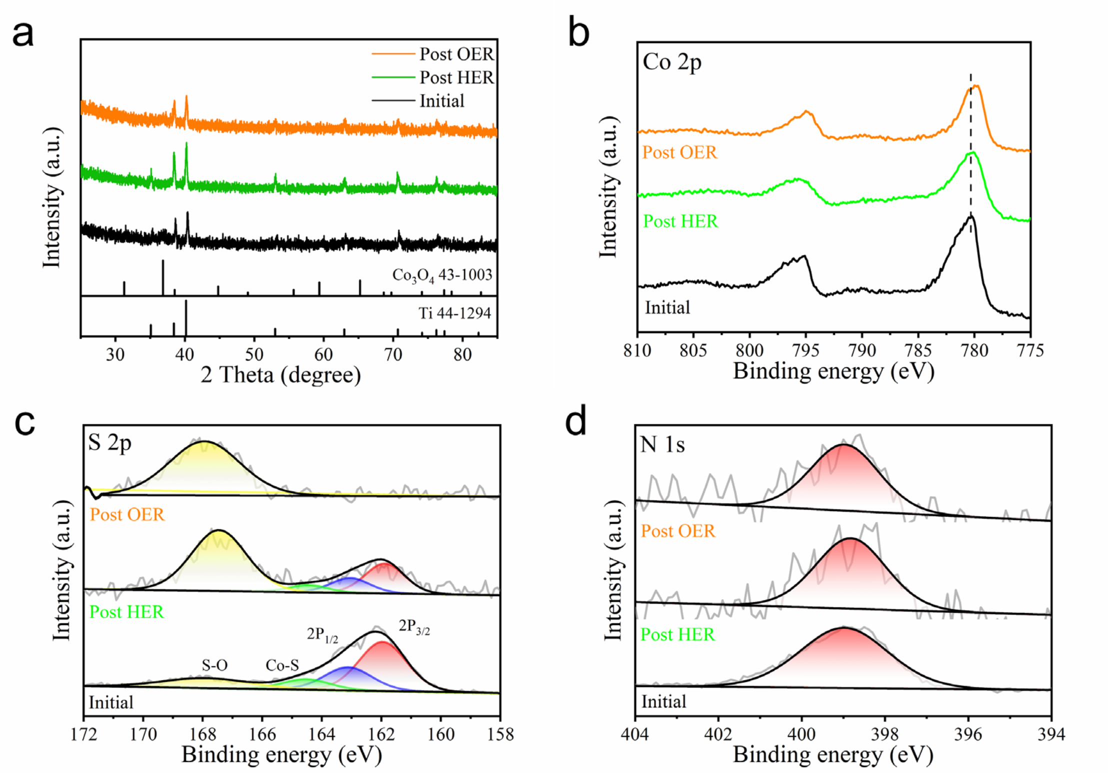

Figure S14. (**a**) XRD, (**b**) Co 2p, (**c**) S 2p and (**d**) N 1s spectra of N/S-Vo-Co_3O_4 before and after HER/OER.

Table S1. Elemental ratios of Co_3O_4 from EDS spectrum.

Element	Atomic Fraction (%)
Co	61.02
O	38.98
S	/
N	/

Table S2. Elemental ratios of N/S-V_O-Co_3O_4 from EDS spectrum.

Element	Atomic Fraction (%)
Co	54.44
O	27.96
S	13.29
N	4.31

Table S3. The relative ratio of Co^{2+} and Co^{3+} in N/S-V_O-Co_3O_4, V_O-Co_3O_4 and Co_3O_4 by Co 2p spectra fitting.

Sample	The ratio of Co^{2+}/Co^{3+}
N/S-V_O-Co_3O_4	0.497
V_O-Co_3O_4	0.534
Co_3O_4	0.507

Table S4. The relative ratio of oxygen vacancies in N/S-V_O-Co_3O_4, V_O-Co_3O_4 and Co_3O_4 by Co 2p spectra fitting.

Sample	The ratio of O_{II}/O_{I}
N/S-V_O-Co_3O_4	0.534
V_O-Co_3O_4	0.681
Co_3O_4	0.333

Table S5. Electrochemical impedance spectroscopy fitting results for N/S-V_O-Co_3O_4, V_O-Co_3O_4, Co_3O_4 during HER at -1.30 V *vs*. SCE.

Sample	R_S (Ω)	R_{CT} (Ω)
N/S-V_O-Co_3O_4	1.53	4.21
V_O-Co_3O_4	1.54	35.83
Co_3O_4	1.57	105.64

Table S6. Electrochemical impedance spectroscopy fitting results for N/S-V_O-Co_3O_4, V_O-Co_3O_4, Co_3O_4 during OER at 0.55 V *vs*. SCE.

Sample	R_S (Ω)	R_{CT} (Ω)
N/S-V_O-Co_3O_4	1.86	2.12
V_O-Co_3O_4	1.91	4.68
Co_3O_4	1.83	6.10

Table S7. Comparison of HER activity for N/S-V_O-Co_3O_4 and recently reported Co-based catalysts

Catalyst	Electrolyte	η_{-10} (mV)	Tafel slope (mV dec^{-1})	Substrate	Reference
N/S-V_O-Co_3O_4	1.0 M KOH	181	79	Ti mesh	This work
P-Co_3O_4	1.0 M KOH	120	51.6	Ti mesh	[58]
C-Co_3O_4	1.0 M KOH	163	89	Ti mesh	[59]
Co_3O_4/MoO_3	1.0 M KOH	195	123	Glassy carbon	[60]
Mo-Co_3O_4	1.0 M KOH	201	198	Ni foam	[61]
$NiCo_2S_4$ NW	1.0 M KOH	210	58.9	Ni foam	[62]
CoSe NCs	1.0 M KOH	228	46	Ti film	[63]
CoS_2	1.0 M KOH	235	129	Ti foil	[64]
Co_3O_4–CuO	1.0 M KOH	288	65	Glassy carbon	[65]
CoS_2@Co_3O_4	1.0 M KOH	320	42	Glassy carbon	[66]
Co_3O_4/MoS_2	1.0 M KOH	348	46	Glassy carbon	[67]

Table S8. Comparison of OER activity for N/S-V_O-Co_3O_4 and recently reported Co-based catalysts.

Catalyst	Electrolyte	η_{10} (mV)	Tafel slope (mV dec^{-1})	Substrate	Reference
N/S-V_O-Co_3O_4	1.0 M KOH	294	71	Ti mesh	This work
C-Co_3O_4	1.0 M KOH	250	54	Ti mesh	[59]
Ni-Co_3Se_4/rGO	1.0 M KOH	284	71	Glassy carbon	[68]
CoP	1.0 M KOH	300	65	Au electrode	[69]
CoN	1.0 M KOH	304	70	Ni foam	[56]
CoS	1.0 M KOH	306	72	Carbon paper	[70]
$NiCo_2O_4$	1.0 M NaOH	320	47	Carbon cloth	[71]
$ZnCo_2O_{4-x}F_x$/CNTs	1.0 M KOH	350	59.2	Glassy carbon	[72]
Co_3S_4	0.1 M KOH+0.6 M NaCl	366	149.6	Glassy carbon	[73]
H-Co_3O_4	1.0 M KOH	380	85	Glassy carbon	[74]
$Mn_{0.5}Ni_{0.5}Co_2O_4$	0.1 M KOH	400	86	Glassy carbon	[75]
